# Tennessee Joins Illinois

**Published:** 1897-06

**Authors:** 


### TENNESSEE JOINS ILLINOIS.

TENNESSEE seems to be in the same situation as Illinois in the recognition of the value of scientific veterinary service. After obtaining an appropriation of $2500 for this work, a salary of $600 is awarded, which will secure only the most meagre service in a State covering so wide a territory, and which frequently has to deal with serious animal scourges. Work of this kind that cannot be done properly, and with some hope of adding to our knowledge of sanitary work, had better be left undone. Equally true is it that a mere political appointment of meat-inspector for Nashville, the ignoring of two competent graduates indorsed by the medical profession of Nashville, and the giving of this place to an uneducated non-graduate, are destined to provoke such severe criticism in these days—not to speak of the inefficient way such work is likely to be done—as to retard and nullify all the efforts made in these directions to free our people from these now well-known dangers, and thus bring down on the heads of these people trusted with authority the execrations of an injured public that they are not likely to enjoy. Strengthen public sentiment in Tennessee, and the battle for better conditions can still be won.

